# Supplementary material for: Using vessels of opportunity for determining important habitats of bottlenose dolphins in Port Phillip Bay, south-eastern Australia
Source: PeerJ. 2024 Oct 30;12:e18400. doi: 10.7717/peerj.18400 (PMC11531264; doi:10.7717/peerj.18400)
Supplement: Supplemental Information 2 [file peerj-12-18400-s002.docx]

**Supplemental Material 2: BioMod2 algorithm settings**

**Default**

**Flexible Discriminant Analysis (FDA)** = method = 'mars', add_args = NULL

**Generalized Additive Modelling (GAM)** = algo = 'GAM_mgcv', type = 's_smoother', k = -1, interaction.level = 0, myFormula = NULL, family = binomial(link = 'logit'), method = 'GCV.Cp', optimizer = c('outer','newton'), select = FALSE, knots = NULL, paraPen = NULL, control = list(nthreads = 1, irls.reg = 0, epsilon = 1e-07, maxit = 200, trace = FALSE, mgcv.tol = 1e-07, mgcv.half = 15, rank.tol = 1.49011611938477e-08, nlm = list(ndigit=7, gradtol=1e-06, stepmax=2, steptol=1e-04, iterlim=200, check.analyticals=0), optim = list(factr=1e+07), newton = list(conv.tol=1e-06, maxNstep=5, maxSstep=2, maxHalf=30, use.svd=0), outerPIsteps = 0, idLinksBases = TRUE, scalePenalty = TRUE, efs.lspmax = 15, efs.tol = 0.1, keepData = FALSE, scale.est = fletcher, edge.correct = FALSE)

**Generalized Boosted Modelling (GBM)** = distribution = 'bernoulli', n.trees = 2500, interaction.depth = 7, n.minobsinnode = 5, shrinkage = 0.001, bag.fraction = 0.5, train.fraction = 1, cv.folds = 3, keep.data = FALSE, verbose = FALSE, perf.method = 'cv', n.cores = 1)

**Multivariate Adaptive Regression Splines (MARS)** = type = 'simple', interaction.level = 0, myFormula = NULL, nk = NULL, penalty = 2, thresh = 0.001, nprune = NULL, pmethod = 'backward')

**User defined**

**Maximum Entropy (MAXENT)** = path_to_maxent.jar = getwd(), memory_allocated = 512, initial_heap_size = NULL, max_heap_size = NULL, background_data_dir = ‘default’, visible = FALSE, linear = TRUE, quadratic = TRUE, product = FALSE, threshold = FALSE, hinge = FALSE, 121qthreshold = 10, beta_1qp = -1.0, beta_categorical = -1.0, betamultiplier = 0.5, defaultprevalence = 0.5
