# Supplementary figures and images for: Using vessels of opportunity for determining important habitats of bottlenose dolphins in Port Phillip Bay, south-eastern Australia
Source: PeerJ. 2024 Oct 30;12:e18400. doi: 10.7717/peerj.18400 (PMC11531264; doi:10.7717/peerj.18400)

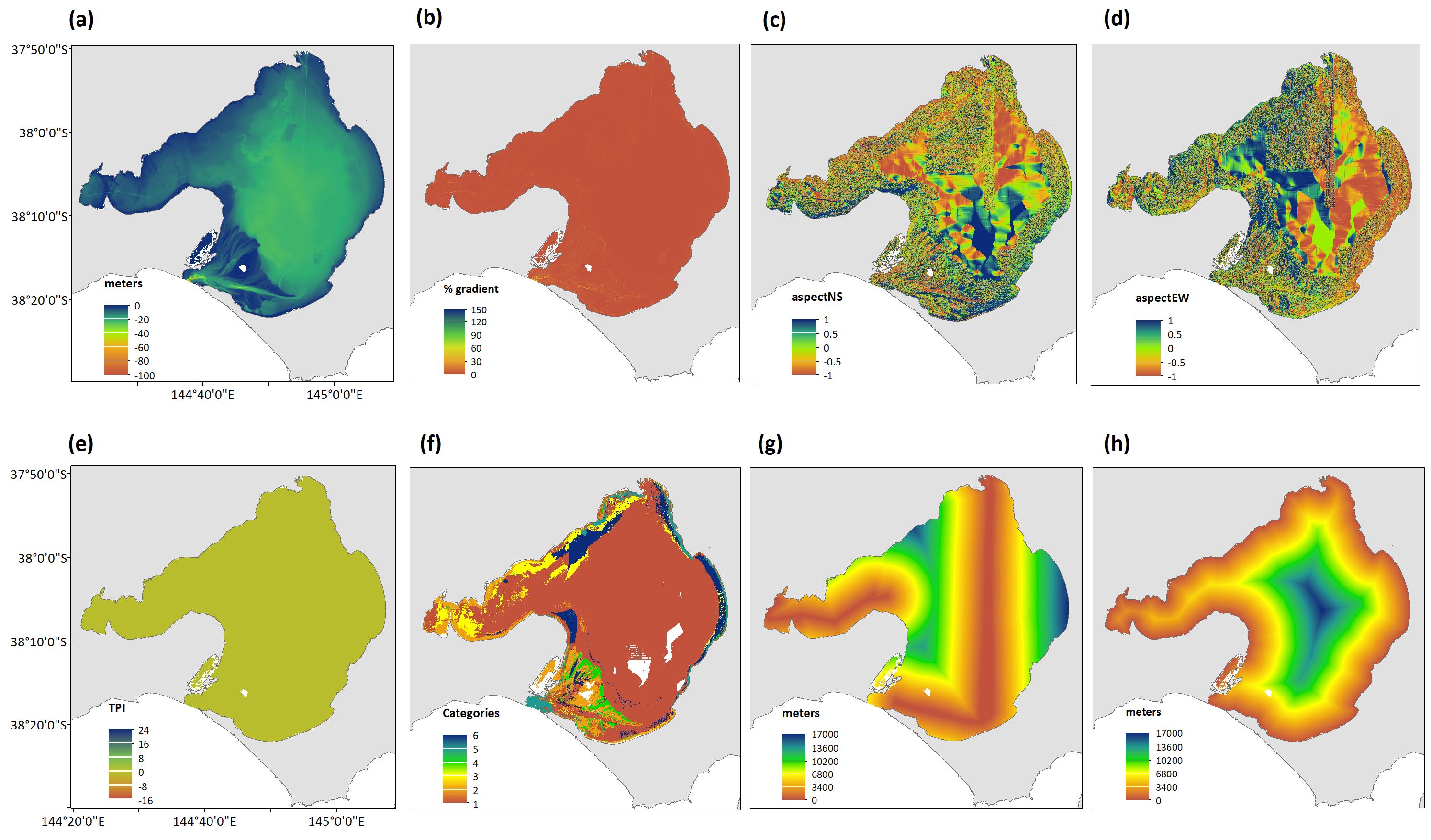

Supplement: Supplemental Information 3 — Variables include: (a) bathymetry (m), (b) seafloor slope (% gradient), (c) seafloor aspect NS and (d) EW, (e) Topographic Position Index (TPI), (f) benthic habitat, (g) Euclidean distance to shipping channels (m) and (h) coastline (m) (map sources: CRCSI (2022); DEECA (2023); Mazor et al. (2023)). Benthic habitat categories are as follows: 1 - sediment; 2 - seagrass beds; 3 - macroalgae on sediment; 4 - biogenic reef; 5 - rocky reef; 6 - sessile invertebrate beds. [file peerj-12-18400-s003.png]
